# Scissors and tweezers: A skin-picking disorder case report

**DOI:** 10.1192/j.eurpsy.2021.1963

**Published:** 2021-08-13

**Authors:** S. Vilas Boas Garcia, N. Fernandes, I. Coelho, R. Costa, R. Durval

**Affiliations:** 1 Unidade Partilhada, Centro Hospitalar Psiquiátrico de Lisboa, Lisboa, Portugal; 2 Serviço De Psiquiatria, Hospital de Santarém, Santarém, Portugal; 3 Centro Hospitalar Psiquiátrico De Lisboa, Centro Hospitalar Psiquiátrico de Lisboa, Lisbon, Portugal

**Keywords:** skin-picking disorder, excoriation disorder, obsessive-compulsive disorders

## Abstract

**Introduction:**

Skin-Picking Disorder (SPD) is psychiatric condition characterized by recurrent and excessive picking of the skin. There are several attempts to stop the behavior and it causes negative consequences such as dermatological complications and functional impairment.

**Objectives:**

The aim of this study is to describe a case report of SPD.

**Methods:**

Data was collected retrospectively from case notes.

**Results:**

A 30 year-old male, married with 2 children, currently on sick leave, was admitted to the Day Hospital at Centro Hospitalar Psiquiátrico de Lisboa (CHPL) with worsen skin-picking behaviour and functional impairment. During childhood the patient would “cut my toe nails the wrong way so that I could fix them”. By adolescence the patient suffered from acne and felt the need to “solve” them and take out the pus. Over the years the skin-picking behaviour spread to other areas of the body, mainly dorsal and chest areas. Before being admitted to the Day Hospital the episodes were daily and had 2-3 hours duration, using scissors and tweezers and evolving his family, asking his wife’s help with picking. He is being treated with fluoxetine 80 mg, risperidone 2 mg and N-acetylcysteine 1200 mg and Cognitive Behavioural Therapy. He is also participating in the Day Hospital activities that include occupational therapy, movement therapy, psychoeducation. After 2 months he has a few 20 minutes episodes per week, spends more time with his children and thinks about coming back to work.

**Conclusions:**

SPD is a severe and debilitating illness that benefits from a multidisciplinary approach.

**Disclosure:**

No significant relationships.

